# Supplementary material for: Fatty acid amide hydrolase drives adult mammary gland development by promoting luminal cell differentiation
Source: Cell Death Discov. 2024 Jan 6;10:12. doi: 10.1038/s41420-023-01788-1 (PMC10771414; doi:10.1038/s41420-023-01788-1)

Fig. 3b

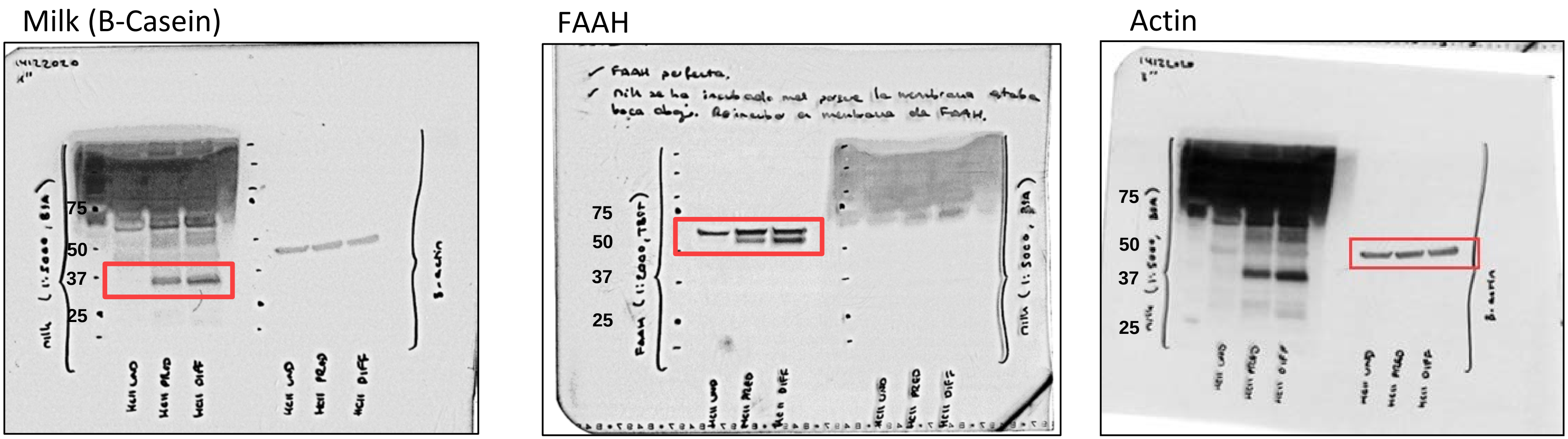

Fig. 3c

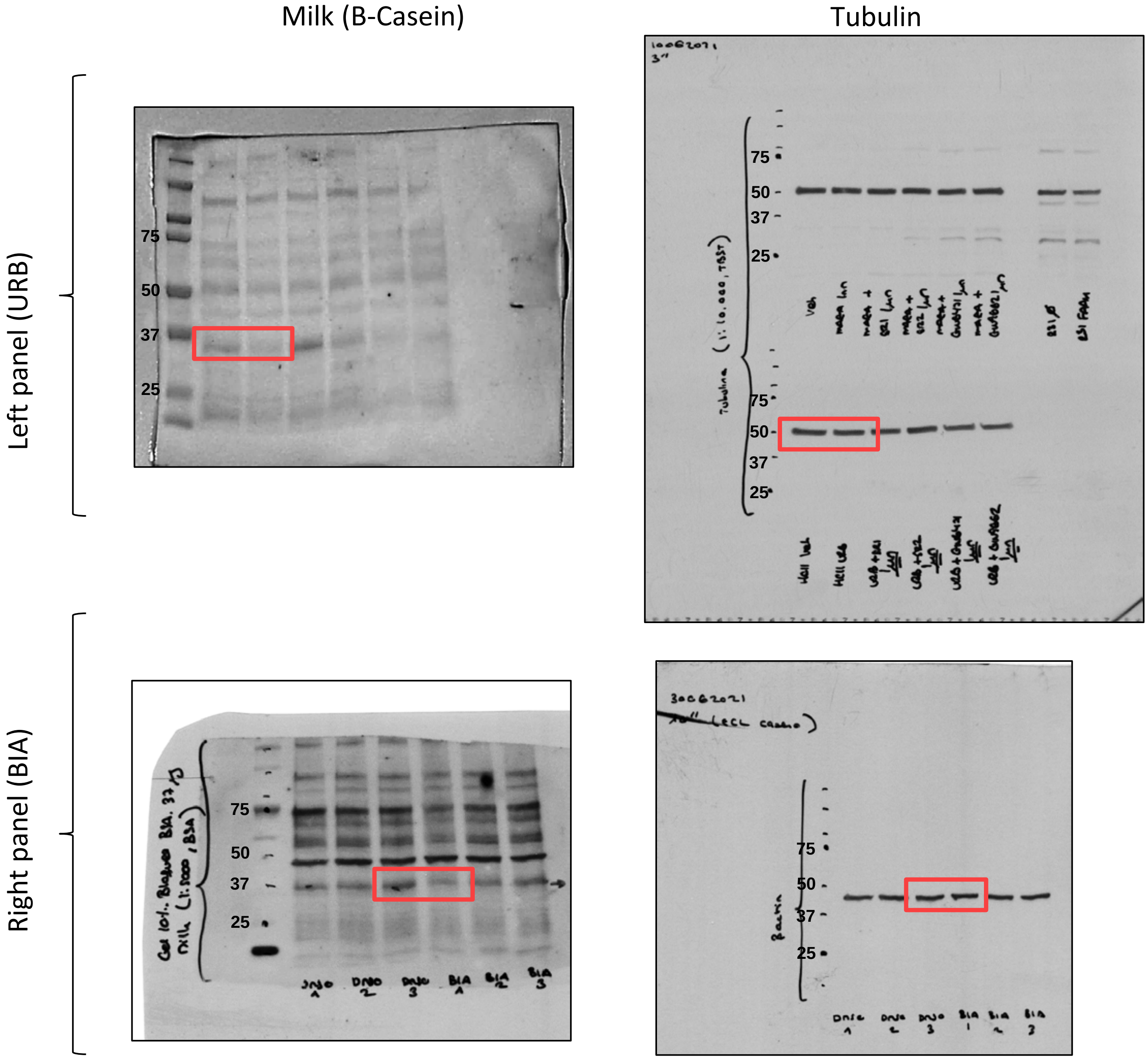
Milk (B-Casein)
Tubulin
Left panel (URB)
Right panel (BIA)
75
50
37
25

Fig. 3d

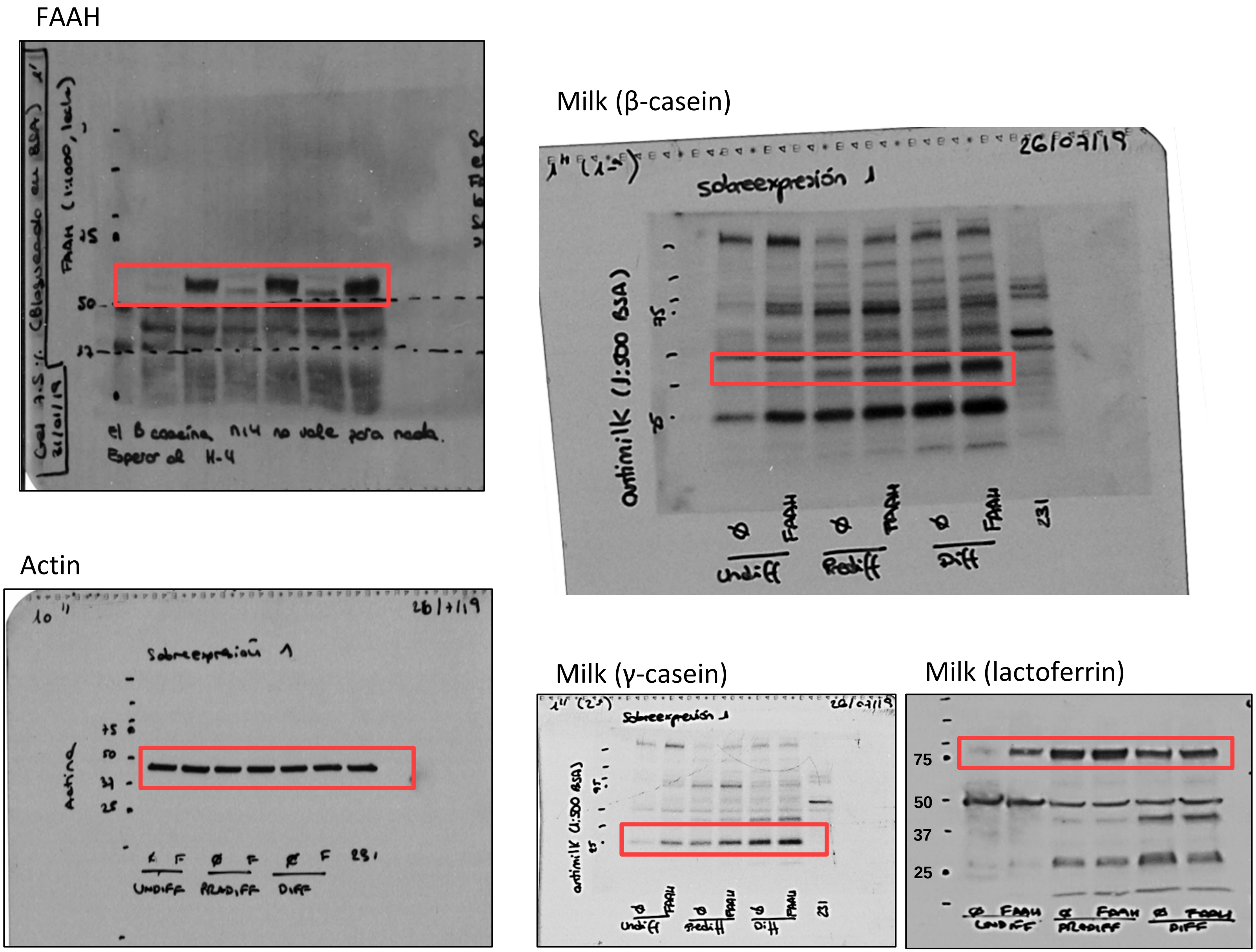
FAAH
Milk (β-casein)
Actin
Milk (γ-casein)
Milk (lactoferrin)
75
50
37
25

Fig. 4a

Milk (β-casein)

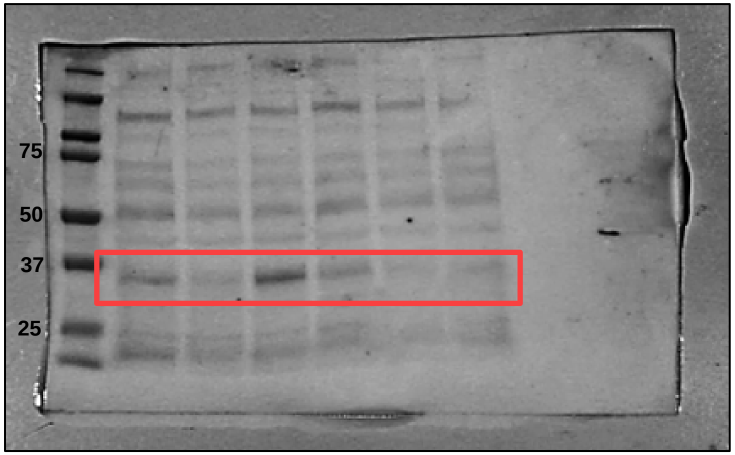

Tubulin

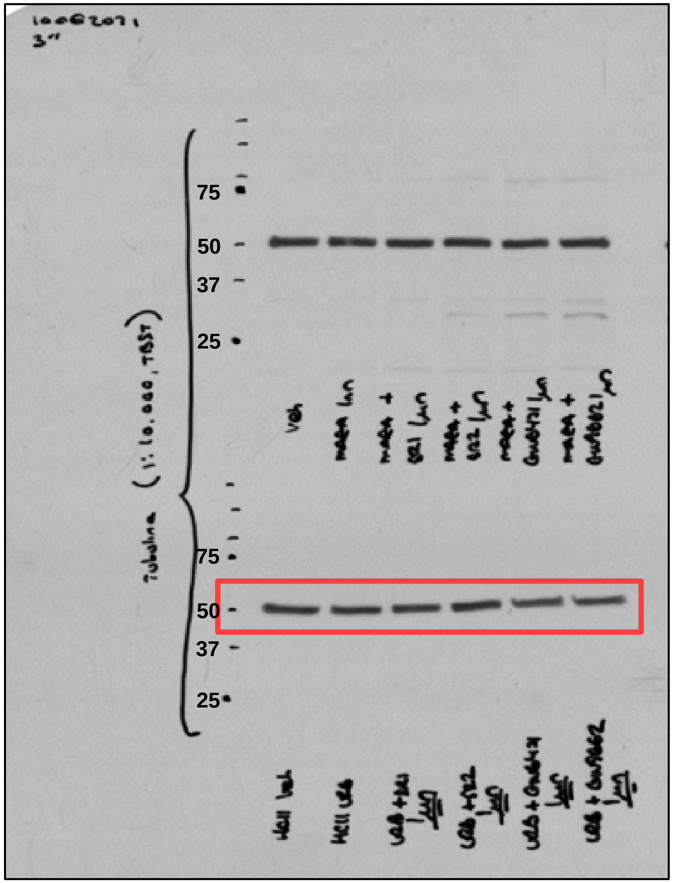

Fig. 4c

Milk (β-casein)

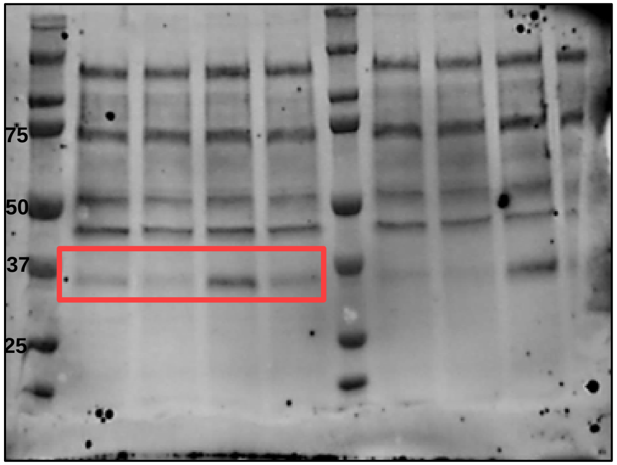

Tubulin

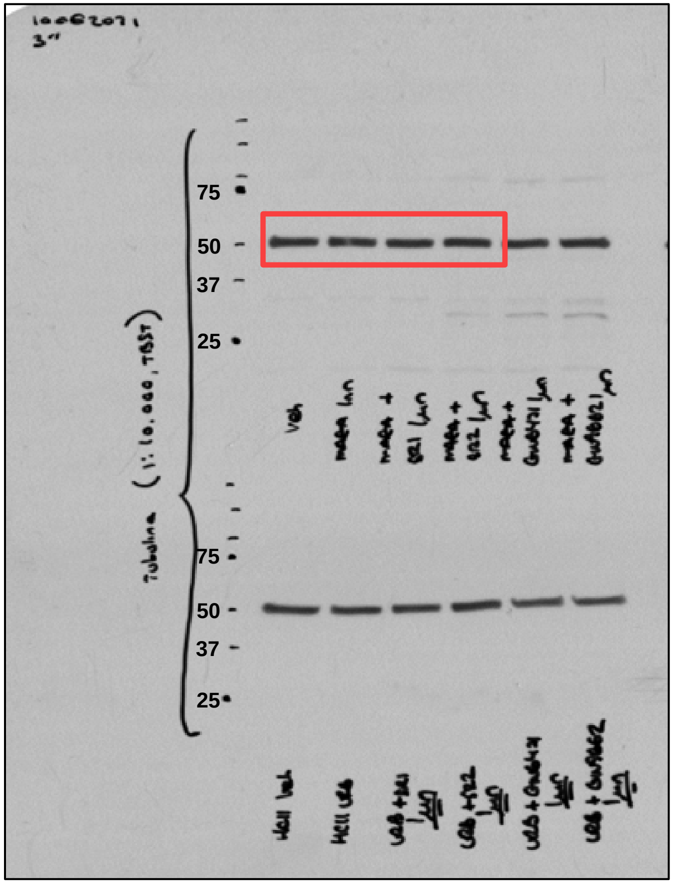

Fig. 4d

Milk (β-casein)

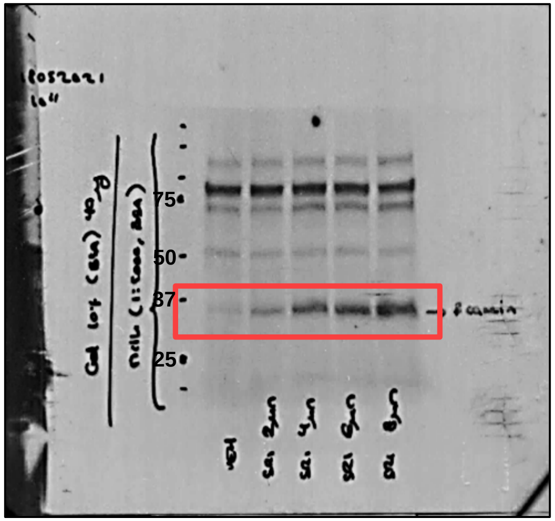

Tubulin

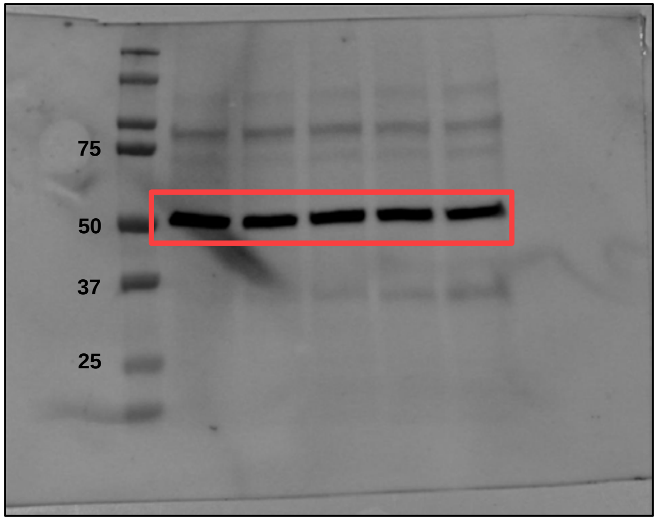

Fig. 6c

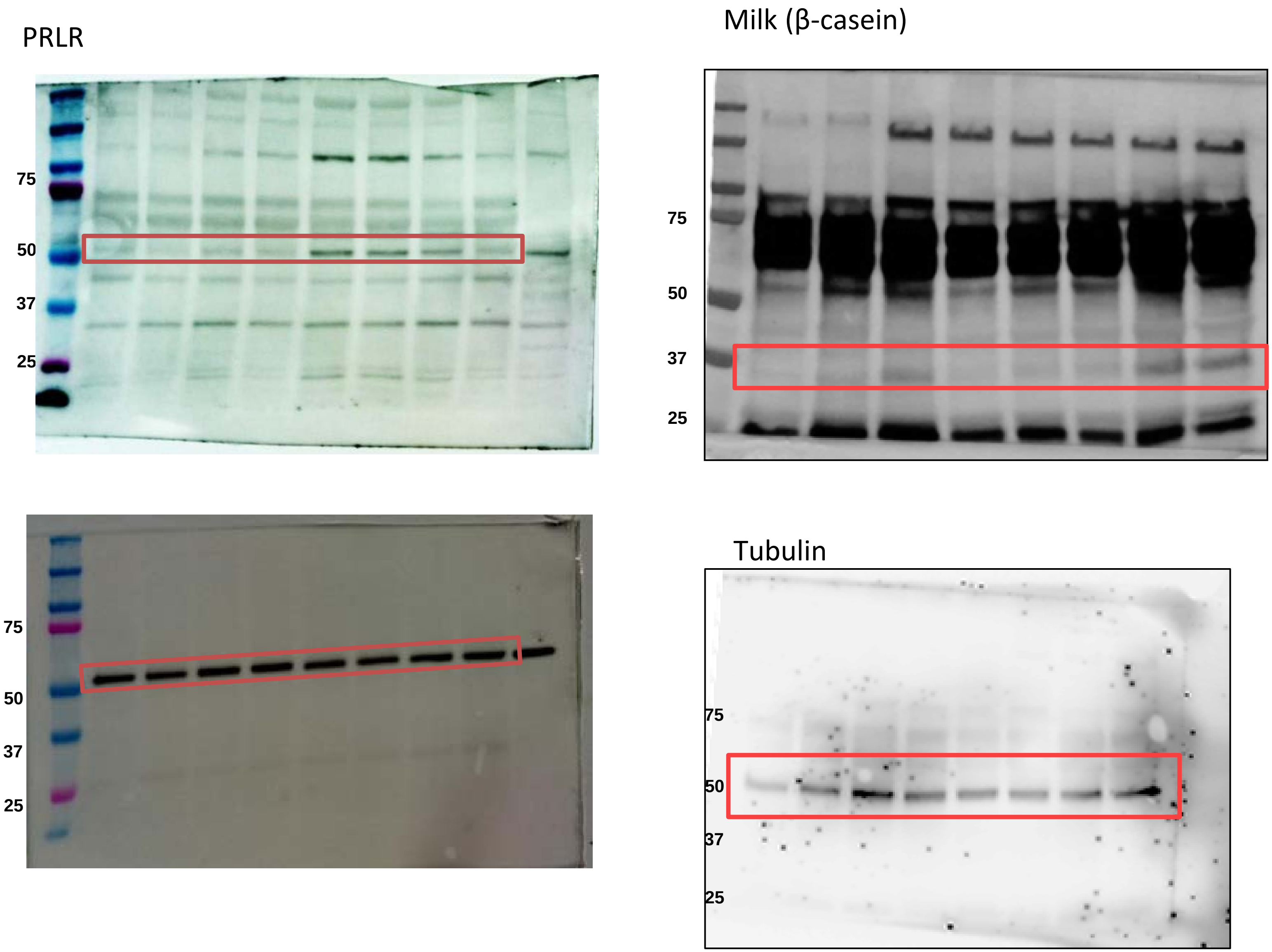
PRLR
75
50
37
25
Milk (β-casein)
75
50
37
25
75
50
37
25
Tubulin
75
50
37
25

Supplementary Fig. 2

FAAH

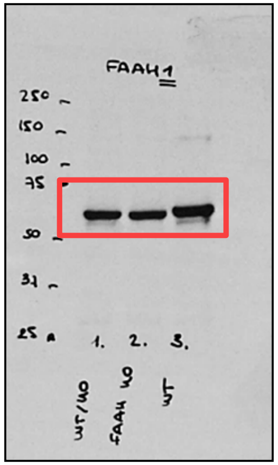

Actin

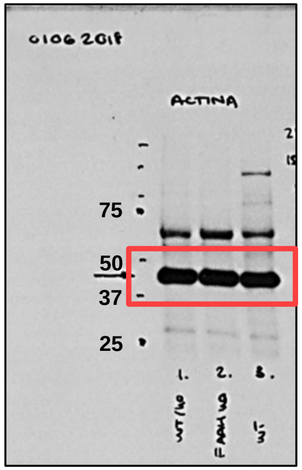

Supplement: Supplementary file 2 — raw western blots [file 41420_2023_1788_MOESM2_ESM.pdf]
